# Comparison and immunobiological characterization of retinoic acid inducible gene-I-like receptor expression in mesenchymal stromal cells

**DOI:** 10.1038/s41598-017-02850-6

**Published:** 2017-06-06

**Authors:** Gordana Raicevic, Mehdi Najar, Hélène Busser, Emerence Crompot, Dominique Bron, Michel Toungouz, Laurence Lagneaux

**Affiliations:** 1 Laboratory of Clinical Cell Therapy, Institut Jules Bordet, Université Libre de Bruxelles, Hôpital Erasme, Université Libre de Bruxelles, Brussels, Belgium; 2 Department of Hematology, Jules Bordet Institute, Hôpital Erasme, Université Libre de Bruxelles, Brussels, Belgium; 3 Department of Immunology-Hematology-Transfusion, Hôpital Erasme, Université Libre de Bruxelles, Brussels, Belgium

## Abstract

Due to their immunomodulatory and regenerative properties, Mesenchymal stromal cells (MSC) have generated major interests in several clinical settings including transplantation and inflammatory diseases. MSC functions can be influenced by their tissue origin. Their microenvironment strongly affects their biology notably through TLR sensing. In this study, we show that MSC isolated from four different sources express another type of cytosolic pathogen recognition receptors known as retinoic acid inducible gene-I (RIG-I)-like receptors (RLR). RLR activation in MSC induces the production of Type I IFN (IFN-β) and Type III IFN (IFN-λ1). The highest producers are adipose tissue(AT)-MSC. We further show that Interferon production is induced through TBK1/IKK-ε signaling and IRF7 phosphorylation. Depending on MSC source, the knockdown of TLR3 and/or RIG-I decreases the MSC response to RLR ligand poly(I:C)/Lyovec. Among the different MSC types, AT-MSCs display the highest sensitivity to viral stimuli as shown by the alteration of their viability after prolonged stimulation. Our work indicates that this could be linked to an increase of pro-apoptotic Noxa expression. Finally, the expression of IDO1 and LIF upon RLR activation indicate the increase of MSC immunomodulatory potential, especially in AT-MSCs. Altogether, these data should be considered when designing MSC-based therapy in clinical settings where inflammation or infection are present.

## Introduction

Classically, inflammatory responses are caused by various factors including microbial infections or tissue injuries. These responses are involved in the generation of adequate immune reactions to detrimental agents contributing to healing processes of damaged tissues. Germline-encoded pattern recognition receptors (PRRs) are major cells sensors of microorganisms through recognition of their pathogen-associated molecular patterns (PAMPs) as well as of damaged cells through recognition of their damage-associated molecular patterns (DAMPs) (reviewed in ref. 1). Up to now, four different classes of PRRs families have been identified including transmembrane proteins such as the Toll-like receptors (TLRs) and C-type lectin receptors (CLRs), as well as cytoplasmic proteins such as the Retinoic acid-inducible gene (RIG)-I-like receptors (RLRs) and NOD-like receptors (NLRs). PRRs are expressed not only by professional immune cells but also by various nonprofessional immune cells. In general, the intracellular signaling cascades triggered by these PRRs lead to transcriptional expression of inflammatory mediators that coordinate the elimination of pathogens and infected cells. The RLR family belongs to the DExD/H-box family of helicases and is composed of RIG-I, melanoma differentiation-associated gene 5 (MDA5), and and laboratory of genetic and physiology 2 (LPG2)^2^. Localized in the cytoplasm, these RLRs recognize the genomic RNA of dsRNA viruses and dsRNA generated as the replication intermediate of ssRNA viruses. The expression of RLRs is greatly enhanced by type I IFN stimulation or virus infection.

Mesenchymal Stromal Cells (MSCs) are nonhematopoietic stem cells with high proliferative, self-renewal, and multilineage capacities. Initially derived from bone marrow (BM), they can be also isolated from other tissues such as adipose tissue (AT), Foreskin (FSK) and umbilical cord (Wharton Jelly’s). They are easily expanded *ex vivo*. Due to their tissue regenerative and immunological properties, MSCs have generated great promises for clinical applications not only in regenerative medicine but also for the treatment of immune mediated diseases thanks to their potent immunomodulatory property. MSC-induced immunomodulation seems to process as a network involving the induction of immune regulatory cells from both the innate and adaptive responses to finally establish a tolerogenic state (reviewed in refs 3 and 4). According to their origin, MSCs may have distinct immunomodulatory effects depending on the regulatory factors produced during interaction with immune cells^5, 6^. MSCs may be seen as sensors and switchers of inflammation. They actively sense their surrounding microenvironment (inflammation vs infection) and modulate, accordingly, the function of host’s immune cells^7^. A better understanding of the interplay between MSCs and both the innate and adaptive immune systems are mandatory for the development of future clinical applications. The communication of MSC’s with the inflammatory microenvironment is an essential part of thair activity process. Depending on its type and intensity, inflammatory stimuli confer to MSCs the ability to suppress or to enhance the immune response highlighting thus a plasticity in the immunomodulatory function of MSCs^8^. Depending on the tissue origin, some TLRs (e.g., TLR3 and TLR4) are differentially expressed by MSCs. Their expression can be strongly modulated by inflammation^9^. Several MSC biological functions such as migration, multilineage potential and immunomodulatory capacity are strongly affected by specific TLR-agonist engagement^10^. In particular, they can be polarized by downstream TLR signaling into two homogenously acting phenotypes^11^. TLR4-primed MSCs (MSC1), mostly producing pro-inflammatory mediators, induce T-lymphocyte activation, while TLR3-primed MSCs (MSC2), mainly expressing immunosuppressive factors, lead to T-cell inhibition.

Although clinical applications of MSCs are promising, long-term observations reveal some complications, including infections which are a major concern in term of morbidity and mortality notably after allogeneic hematopoietic stem cell transplantation^12, 13^. An additional concern lies in the MSC susceptibility to viral infections, raising a safety issue after *ex vivo* expansion before clinical use. Infected MSCs may also present disclose impaired immunosuppressive and antimicrobial functions undermining their clinical efficacy. Inflammation and infection are known to be major events triggering GVHD after allogeneic stem cell transplantation^12, 14^. Differential effects of MSCs on immune cells in response to viral infection or infectious agents were already reported. It is important to take into account the dual effect of MSCs on the antiviral immune response when an infection might be present^15^. During an infectious process, TLRs and RLRs have common and distinct features. In contrast to TLR3, which sense viral dsRNA present in extracellular compartment or in endosomes, the intracellular dsRNA is detected by cytosolic RLR. It has been shown that MDA5 recognizes long dsRNA, such as synthetic poly(I:C) greater than 1000 nucleotides, whereas RIG-I receptor for the recognition of viral infections requires blunt ended 5′ tri-phosphate dsRNA (reviewed in ref. 1)^16^. The role of LGP2 in cytosolic RNA sensing is still unclear but some reports suggest that it functions as a positive regulator of RIG-I and MDA5-mediated antiviral responses^17^. Both RIG-I and MDA5 induce antiviral response through the activation of IRF3/7 and NF-kB transcription factors leading to upregulation of gene expression and production of type I IFN, type III IFN as well as pro-inflammatory cytokines such as IL-6 and IL-8^15^. Type I IFNs are rapidly induced and play an essential role in defense against viral infection at any stage of the virus life cycle. By feeding back in an autocrine and paracrine manner, IFNα/β induce an anti-viral state in surrounding cells by inducing transcription of genes involved in apoptosis, anti-growth, and innate and adaptive immune cell activation. Apoptosis has been known as one of the important RLR activation–mediated antiviral responses in many cell types. For example, activation of RLRs in melanoma cells might couple to the induction of caspase-dependent apoptotic response via the BH3-only protein Noxa^17^. The induction of this pro-apoptotic molecule initiates intrinsic apoptosis pathway (via mitochondrial membrane permeability)^18^. Moreover, RLRs activation can induce the extrinsic apoptotic pathway by upregulating the expression of TRAIL^19, 20^. Recently it has been shown that mice MSCs express functional RIG-I and MDA-5 receptors, and that their activation induce apoptosis of MSCs, independently of IFN-β^21^. Recently, Yu *et al*. reported functional roles of PRR in human adipose-derived MSC mediating antivirus responses^22^.

In the present study, we characterize the expression of RLRs in human MSCs from different tissue origins. We further describe the impact of RLRs activation on the MSC immunobiology including phenotype, survival, signaling pathways and secretome. These data could help designing new new MSC-based therapeutic strategies particularly in inflammatory and infectious contexts.

## Results

### Expression of RIG-I and MDA-5 in MSCs isolated from different tissues

**Figure 1:**
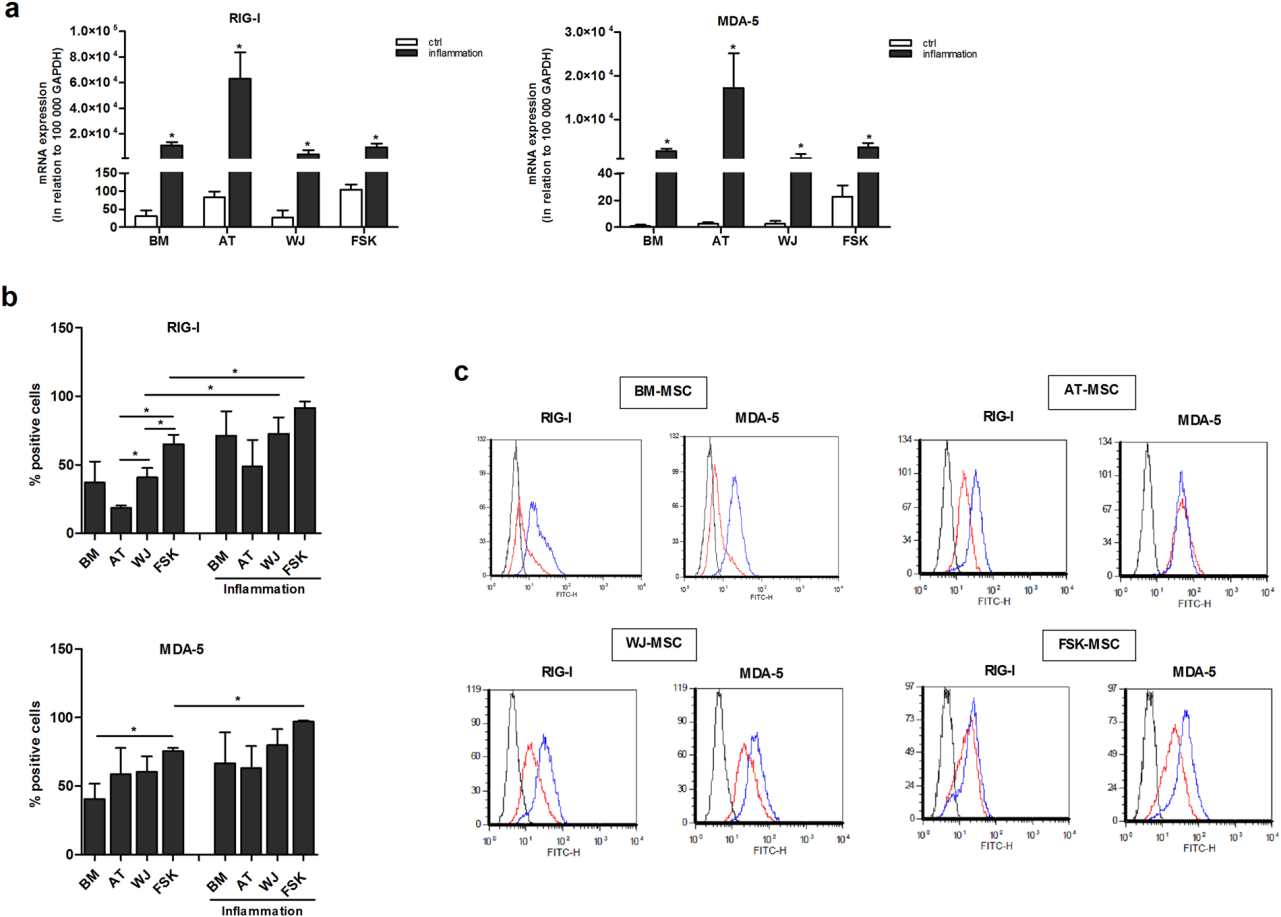
Inflammation influences the expression of RIG-I and MDA-5 receptors in MSCs isolated from different tissues. (**a**) Relative expression of RIG-I and MDA-5 in BM-, AT-, WJ- and FSK-MSCs was measured by real-time PCR. Different MSC were incubated in the presence or absence of an inflammatory cytokine cocktail for 24 h. Then total RNA was extracted and the expression of RIG-I and MDA-5 mRNA was evaluated by quantitative PCR and normalized for the housekeeping gene GAPDH expression. Mean values of mRNA relative quantity ± SEM of three independent experiments are represented; (**b**) Expression of RIG-I and MDA-5 at the protein level assessed by flow cytometry. The cells were incubated in the presence or not of the inflammation for 24 h. The cells were then fixed, permeabilized and labeled with primary rabbit anti-human polyclonal antibodies against RIG-I and MDA-5. The primary labeling was visualized with a secondary goat anti-rabbit IgG FITC-conjugated antibody. Data are presented as the mean percentage ± SEM of positive cells. n = 3, *p < 0.05; (**c**) Representative flow cytometry histograms of RIG-I and MDA-5 expression in the absence (red line) or presence (blue line) of inflammation in four different MSC types. Black line represents negative control.

The expression of RIG-I and MDA-5 was evaluated in MSCs isolated from BM, AT, WJ and FSK using real time PCR. All four types of MSCs expressed RIG-I while the expression of MDA-5 was quite low except for FSK-MSCs (Fig. 1a). In the presence of an inflammatory environment, the mRNA expression of both receptors was significatively increased.

Flow cytometry analysis have further confirmed the presence of these two receptors in all MSC types. The highest expression of RIG-I was in FSK-MSCs while the lowest one was in AT-MSCs. In a line with mRNA data, the inflammation increased the expression of this receptor in all MSC types. MDA-5 was highly expressed in FSK-MSCs, lower expression was observed in AT- and WJ-MSCs and the lowest one in BM-MSCs. The inflammation increased the expression of MDA-5 only in FSK-MSCs (Fig. 1b and c).

### Poly(I:C)/LyoVec induces cytokine expression in different MSC types

**Figure 2:**
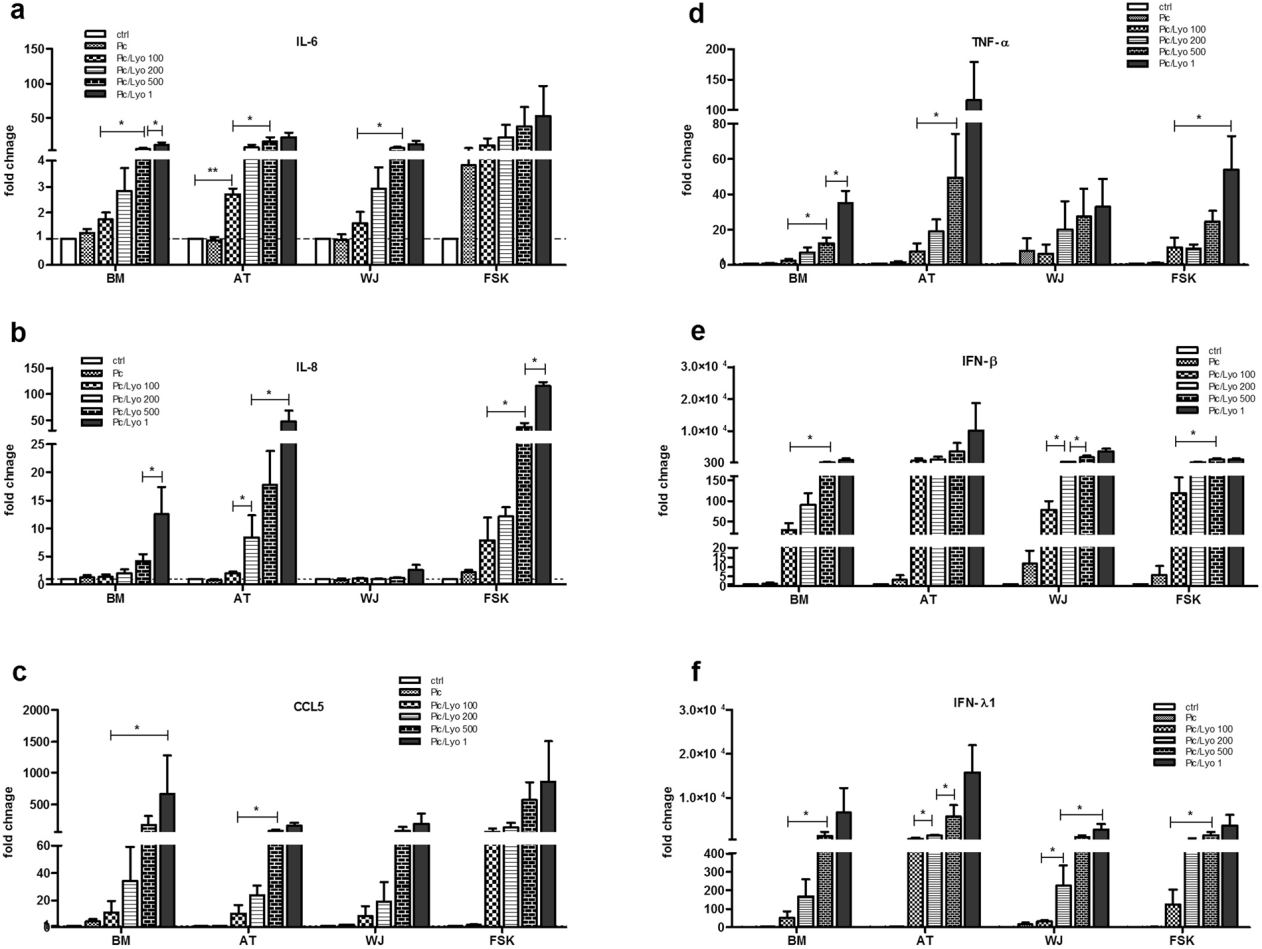
Activation of MDA-5 and RIG-I receptors in MSC increases the mRNA expression of cytokines and interferons. MSC isolated from BM, AT, WJ and FSKs were incubated in the medium in the presence of 25 µg/ml Poly(I:C) or were transfected with 100 ng/ml, 200 ng/ml, 500 ng/ml and 1 µg/ml of Poly(I:C)/LyoVec for 24 h. Then mRNA was extracted and the expressions of (**a**) IL-6, (**b**) IL-8, (**c**) CCL5, (**d**) TNF-α, (**e**) IFN-β and (**f**) IFN-λ1 was evaluated by quantitative PCR and normalized for the housekeeping gene GAPDH expression. The results are expressed as relative mRNA fold change ± SEM (n = 3), *p < 0.05. Abbreviations: ctrl, control; Pic, Poly(I:C); Pic/Lyo, Poly(I:C)/LyoVec.

The functionality of the expressed RIG-I/MDA-5 receptors was tested using different concentrations of the ligand Poly(I:C) in complex with the transfection reagent LyoVec. The different types of MSCs responded to RIG-I/MDA-5 activation by up-regulating the expression of IL-6, IL-8, CCL5, TNF-α, IFN-β and IFN-λ1 and this increase of RNA expression was dose-dependent (Fig. 2a–f). When comparing the different MSC types, FSK-MSC had the strongest response as even at the lowest Poly(I:C)/LyoVec concentration the fold change for IL-6, IL-8 and CCL5 RNA expression was higher than in the MSCs from the other sources. While the influence of different concentrations of Poly(I:C)/LyoVec ligand on the expression of IL-6 was comparable between AT-, BM- and WJ-MSCs, the IL-8 expression was increased in BM-MSCs only at two higher ligand concentrations but was not increased at all in WJ-MSCs (Fig. 2a–c).

However, the RIG-I/MDA-5 activation in AT-MSCs had the strongest impact on IFN-β and IFN-λ1 expressions as compared with the other MSC types as both interferons were induced even at the lowest ligand concentrations (Fig. 2e,f). Moreover, RIG-I/MDA-5 activation in AT-MSCs had also the strongest impact on the TNF-α expression compared with the other types of MSCs (Fig. 2d).

**Figure 3:**
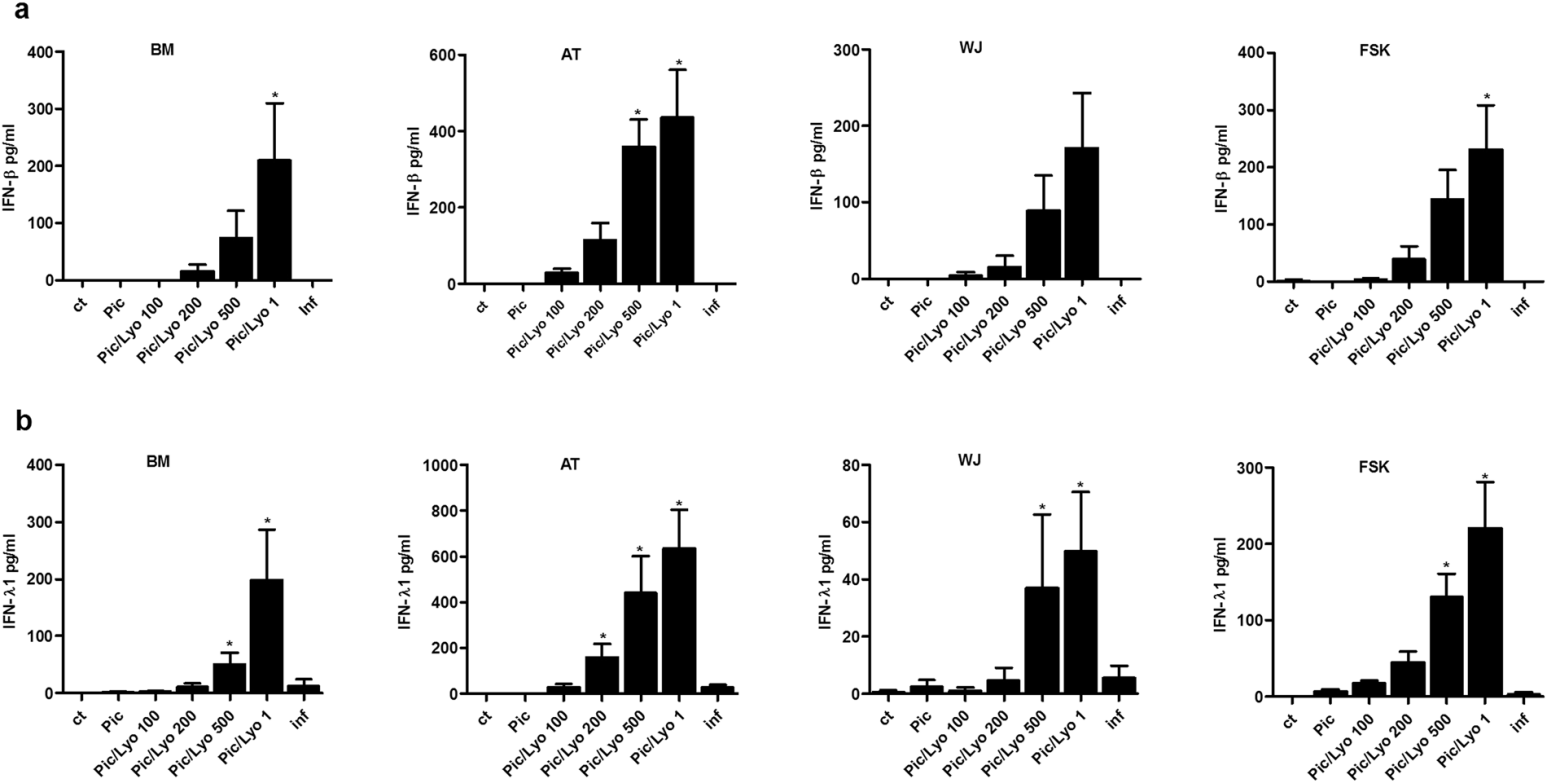
Activation of MDA-5 and RIG-I receptors in MSCs triggers the production of interferons. MSC isolated from BM, AT, WJ and FSK were incubated in the medium in the presence of 25 µg/ml Poly(I:C) or were transfected with 100 ng/ml, 200 ng/ml, 500 ng/ml and 1 µg/ml of Poly(I:C)/LyoVec or were incubated in the presence of an inflammatory cytokine coktail for 24 h. Then supernatants were collected and the (**a**) IFN-β and (**b**) IFN-λ1 production was measured by ELISA. n = 3, *p < 0.05. Abbreviations: ctrl, control; Pic, Poly(I:C); Pic/Lyo, Poly(I:C)/LyoVec; Inf, Inflammation.

None of the MSCs tested in our experiments produced interferons constitutively. However, when transfected with Poly(I:C)/LyoVec, BM-, WJ-, AT- and FSK-MSCs secreted IFN-β and IFN-λ1 but not IFN-α as confirmed by ELISA. The production of both interferons was ligand dose-dependent (Fig. 3). These results corresponded to those obtained by real time PCR. The RIG-I/MDA-5 activation triggered the production of IFN-β and IFN-λ1 by AT-MSCs even at the very low ligand concentration. MSCs from the three other sources had comparable production of IFN-β while WJ-MSCs produced very low levels of IFN-λ1 even at the highest concentration (Fig. 3a and b).

Poly(I:C) added in the medium, activating TLR3, did not trigger the production of IFN-β and IFN-λ1 in any MSC type. The presence of inflammation as well did not influence interferon production by studied MSC.

### Involvement of TLR3 and RIG-I in MSC response to Poly(I:C)/Lyovec stimuli

**Figure 4:**
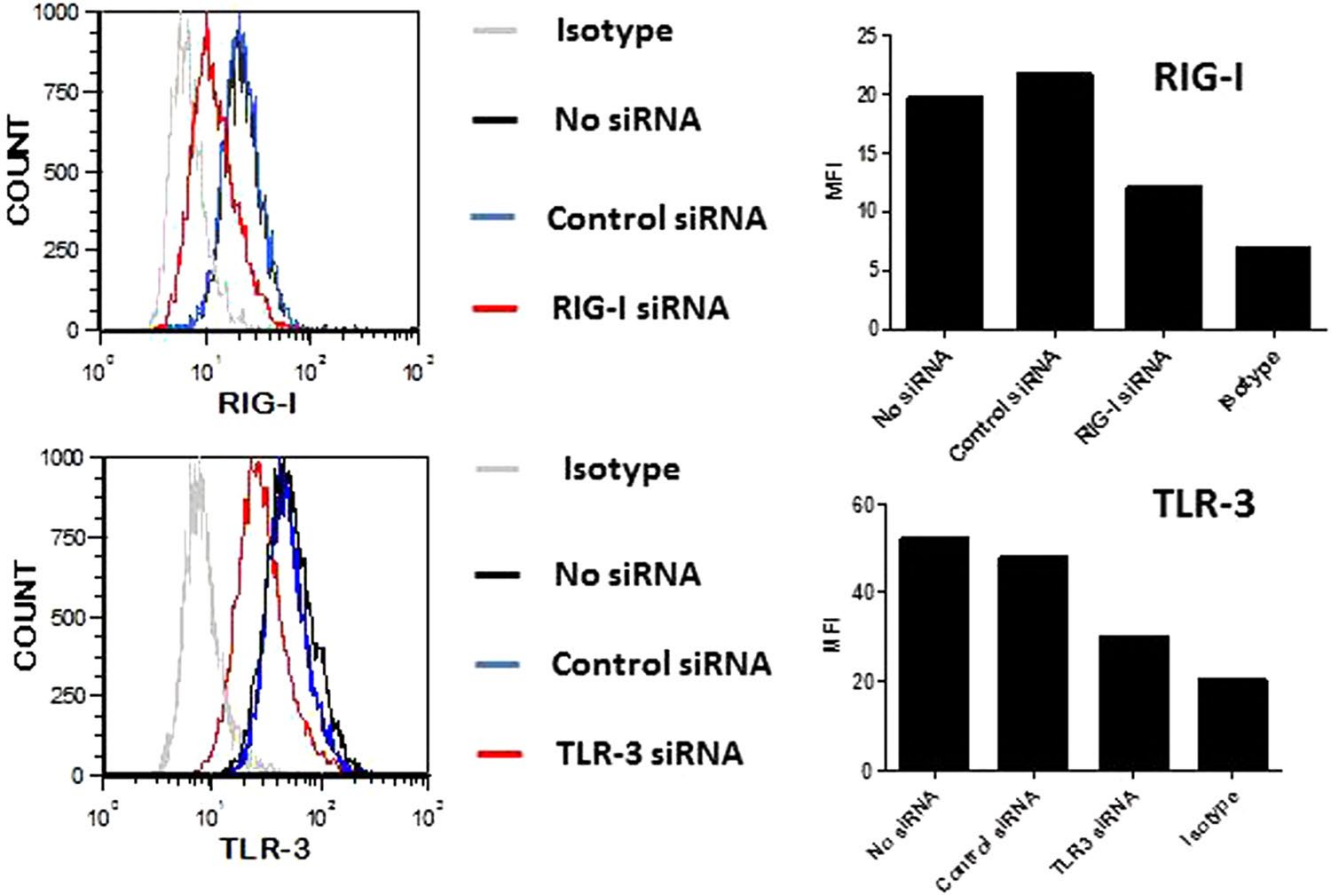
Knockdown of TLR3 and RIG-I. We confirmed by flow cytometry the reduced expression of TLR3 and RIG-I after using specific siRNA in all MSC types. Representative experiment of the transfection of BM-MSCs with control siRNA, RIG-I siRNA and TLR3 siRNA. The efficency of transfection has been confirmed for all MSC sources.

**Figure 5:**
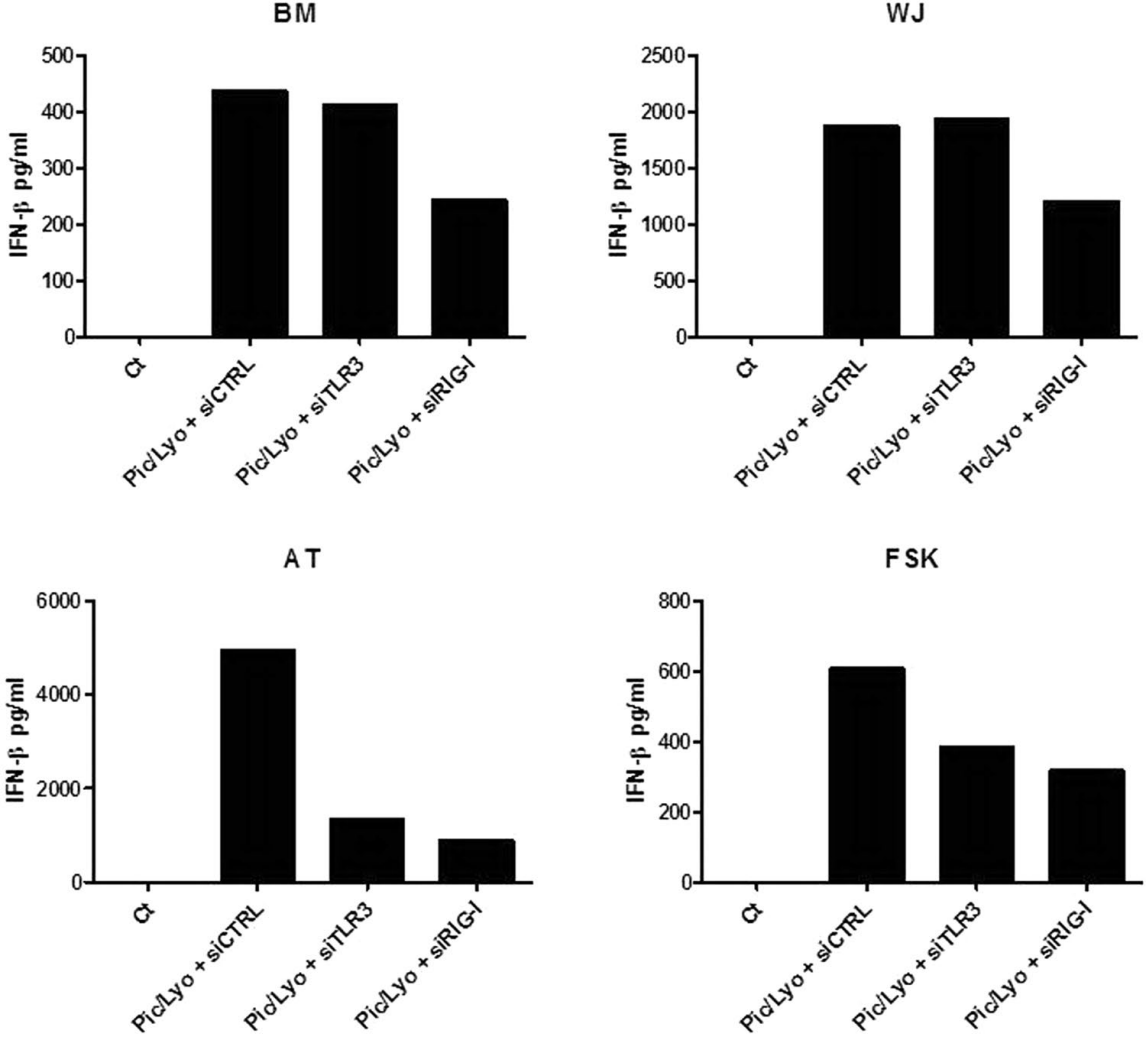
Involvement of RIG-I and TLR3 in poly(I:C)/LyoVec-induced IFN-β release. MSC from different sources were transfected with control, TLR3 and RIG-I siRNA and after 24 h were stimulated with poly(I:C)/LyoVec at 1 µg/ml. The levels of IFN-β in condioned media were measured by ELISA.

To determine TLR3 and RIG-I implication in Poly(I:C)/Lyovec induced MSC response, we employed the siRNA approach. For each MSC source, TLR3 and RIG-I were silenced using specific small interfering RNA (siRNA). A siRNA targeting a scrambled sequence was used as control. After transfection with siRNA, the expression of TLR3 and RIG-I was evaluated by flow cytometry. We confirmed by this method the reduced expression of TLR3 and RIG-I after using specific siRNA in all MSC types. As observed on Fig. 4, the control siRNA didn’t modify the expression of these two receptors. In contrast, the expression of TLR3 and RIG-I was downregulated with the specific siRNA. The mean fluorescence intensity (MFI) of each receptor was reduced by more than 50%. The RIG-I siRNA reduced the Poly(I:C)/LyoVec-induced expression of IFN-β in all MSC sources (Fig. 5). However the TLR3 siRNA decreased the production of IFN-β only in two MSC types, AT and FSK. These results suggest that Poly(I:C)/LyoVec acts through RLR rather than through TLR3 in MSCs. Nevertheless, depending on source, TLR3 can be also implicated in this response.

### Activation of RLR induces the production of interferons through TBK1/IKK-ε and IRF7 phosphorylation

**Figure 6:**
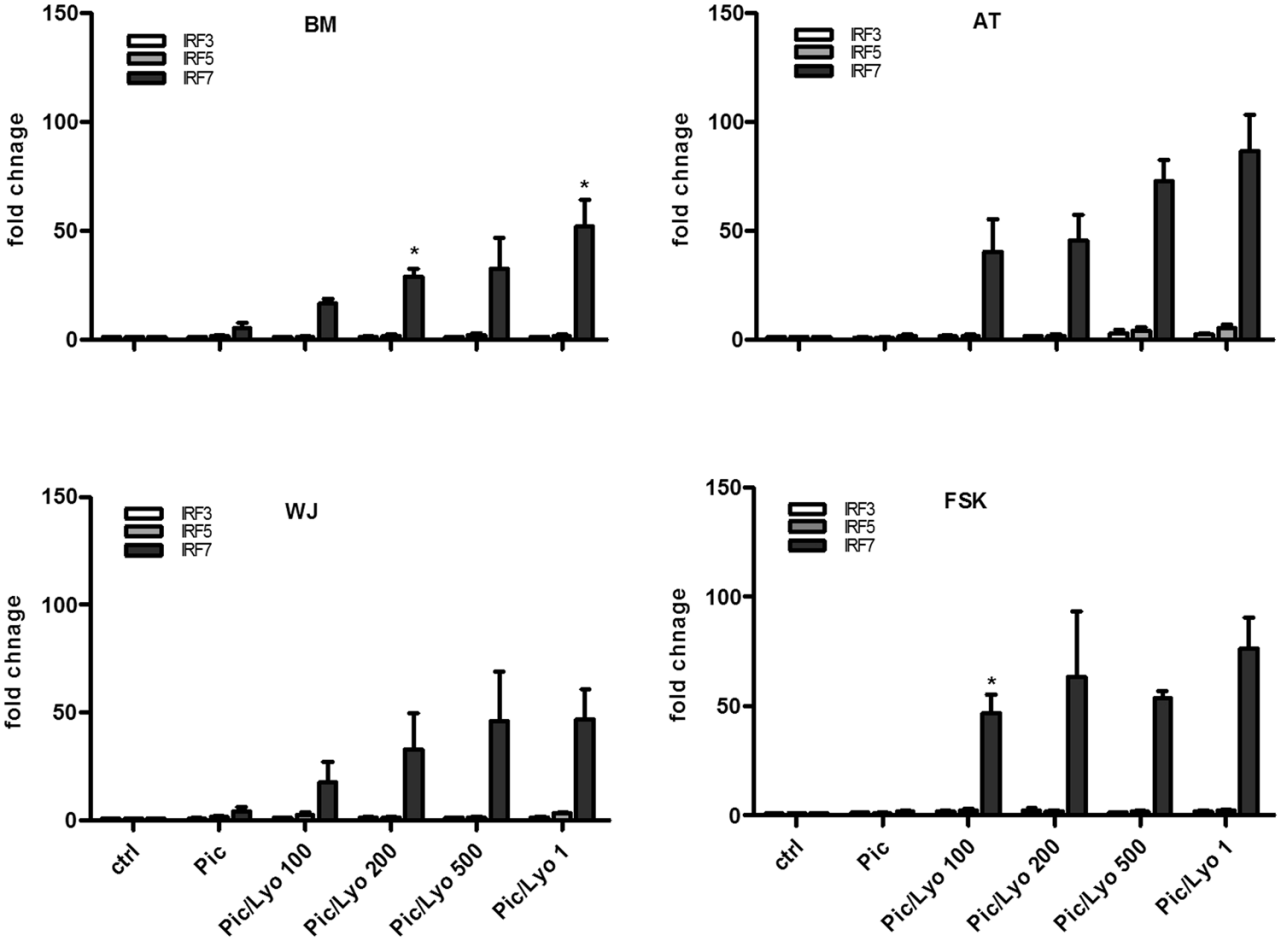
Activation of MDA-5 and RIG-I receptors in MSCs increases the expression of IRF7. MSCs isolated from BM, AT, WJ and FSK were incubated in the medium in the presence of 25 µg/ml Poly(I:C) or were transfected with 100 ng/ml, 200 ng/ml, 500 ng/ml and 1 µg/ml of Poly(I:C)/LyoVec for 24 h. Then mRNA was extracted and the expressions of IRF3, IRF5 and IRF7 were evaluated by real time PCR. The results are expressed as relative mRNA fold change ± SEM (n = 3), *p < 0.05. Abbreviations: ctrl, control; Pic, Poly(I:C); Pic/Lyo, Poly(I:C)/LyoVec.

**Figure 7:**
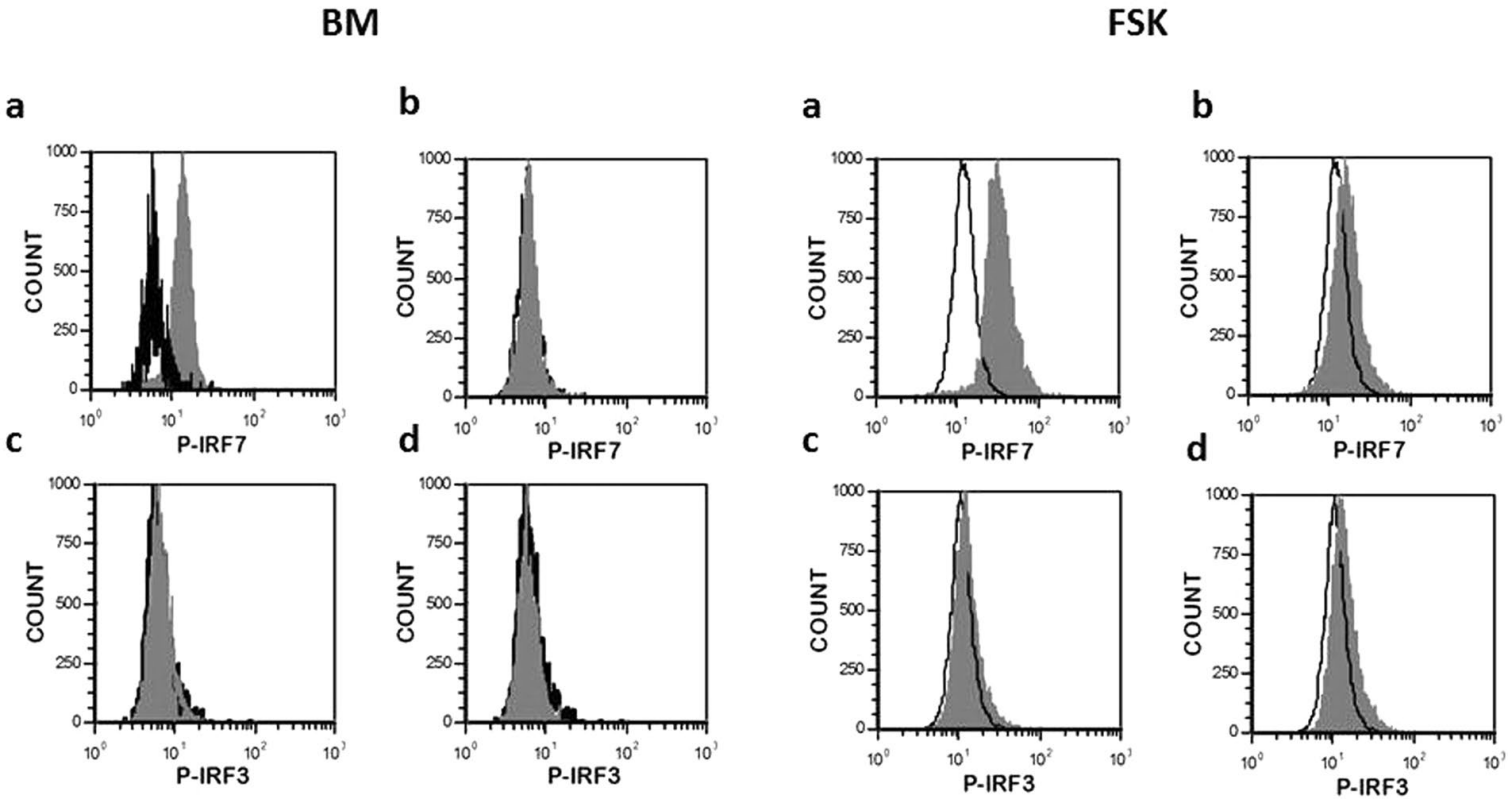
Poly(I:C) transfection induces the phosphorylation of IRF7 but not IRF3 in MSCs. Treatment of MSCs with Amlexanox, an inhibitor of the protein kinases TBK1 and IKK-ε inhibited the IRF7 activation. The phosphorylation of IRF3 and IRF7 was evaluated by flow cytometry after cell fixation and permeabilization. Representative experiments for BM- and FSK-MSCs are shown but similar results were obtained for other sources. (**a**) and (**c**) untreated MSCs (**b**) and (**d**) Amlexanox-treated MSCs.

**Figure 8:**
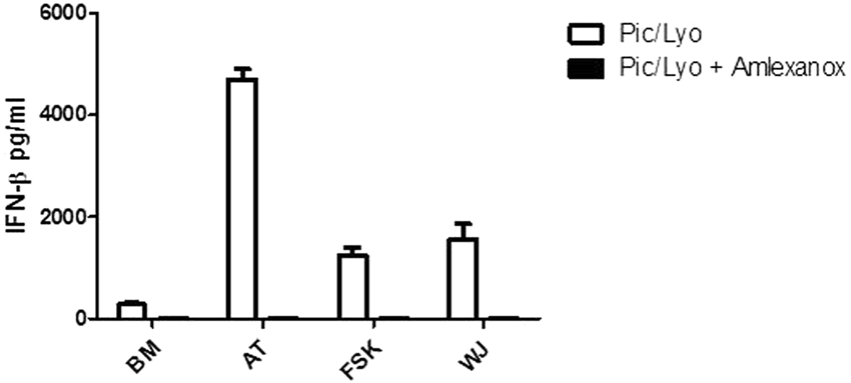
The treatment of MSCs with Amlexanox inhibited the release of IFN-β after Poly (I:C) transfection. MSCs were pretreated with Amlexanox at 100 µM for 1 h and then transfected with Poly(I:C)/Lyovec used at 1 µg/ml.

Our qPCR experiments showed that interferon expression was linked to the presence of the IRF7 transcription factor as its expression was increased in all MSC types when transfected with Poly(I:C) (Fig. 6). To confirm the potential activation of IRF7, we evaluated the phosphorylation of IRF7 and IRF3 in response to Poly(I:C)/LyoVec by flow cytometry. The obtained results showed that this ligand clearly induced the phosphorylation of IRF7 (Fig. 7) while the phosphorylation of IRF3 was undetectable. Moreover we evaluated the effect of Amlexanox, an inhibiteur of TBK1 and IKK-ε, upstream molecules of IRF7, on the phosphorylation of IRF7. MSC were pre-treated for 1 h with Amlexanox at 100 µM and then treated with Poly(I:C)/LyoVec for 24 h. In the presence of this inhibitor, we didn’t observed the phosphorylation of IRF7 (Fig. 7). Amlexanox inhibited also the release of IFN-β by all MSC sources in response to Poly(I:C)/LyoVec demonstrating the involvement of TBK1/IKK-ε and IRF7 in Poly(I:C)/LyoVec triggered IFN-β production (Fig. 8).

### Poly(I:C)/LyoVec causes the upregulation of all the three RLR and TLR3 mRNA expression

**Figure 9:**
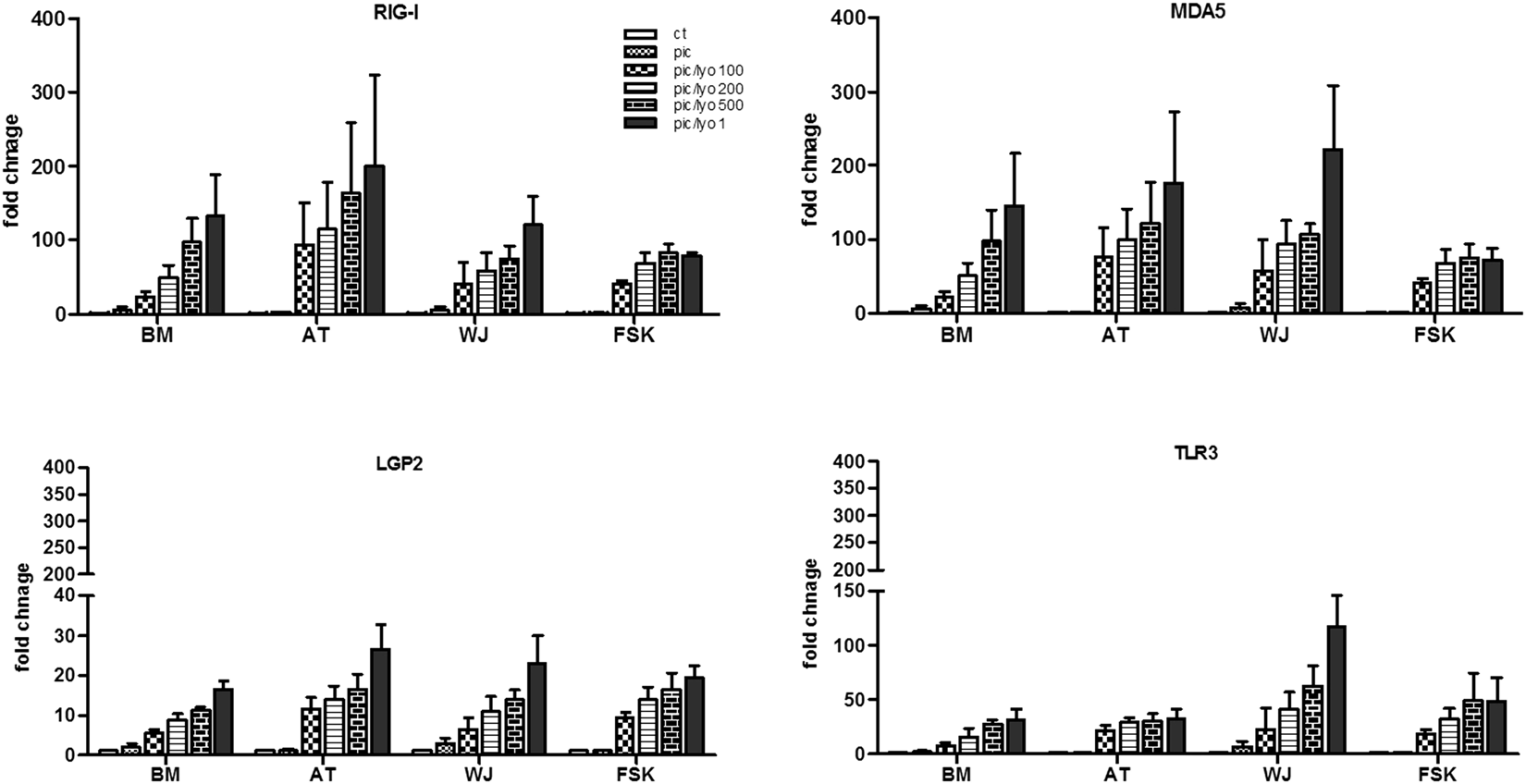
MSC transfection with Poly(I:C)/LyoVec increases the expressions of RLR and TLR3. MSC isolated from BM, AT, WJ and FSK were incubated in the medium in the presence of 25 µg/ml Poly(I:C) or were transfected with 100 ng/ml, 200 ng/ml, 500 ng/ml and 1 µg/ml of Poly(I:C)/LyoVec for 24 h. Then mRNA was extracted and the expression of RIG-I, MDA5, LGP2 and TLR3 was evaluated by real time PCR. The results are expressed as relative mRNA fold change ± SEM (n = 3). Abbreviations: ctrl, control; Pic, Poly(I:C); Pic/Lyo, Poly(I:C)/LyoVec.

To test the influence of TLR3 and RIG-I/MDA5 ligands on the expression of these receptors, we performed real time PCR. After 24 h of cell incubation in the presence of Poly(I:C)/LyoVec, a strong concentration-dependent increase of RIG-I and MDA5 mRNA expression in BM-, AT- and WJ-MSCs was observed. However, RIG-I and MDA5 mRNA expression in FSK-MSCs reached the maximum (70-fold) at a concentration of 200 pg/ml. Higher concentrations did not have any further impact on further receptor transcription (Fig. 9). The expression of LGP2 was also increased in the presence of dsRNA although to a lesser extent than in the case of the two other RLR, with a maximal 26-fold change for AT-MSCs at the highest concentration (Fig. 9). Our result showed that Poly(I:C) transfection of different MSCs increased the expression of TLR3 receptors with the highest influence on WJ-MSCs (Fig. 9).

### RIG-I/MDA-5 activation influences IDO1 and LIF expression

**Figure 10:**
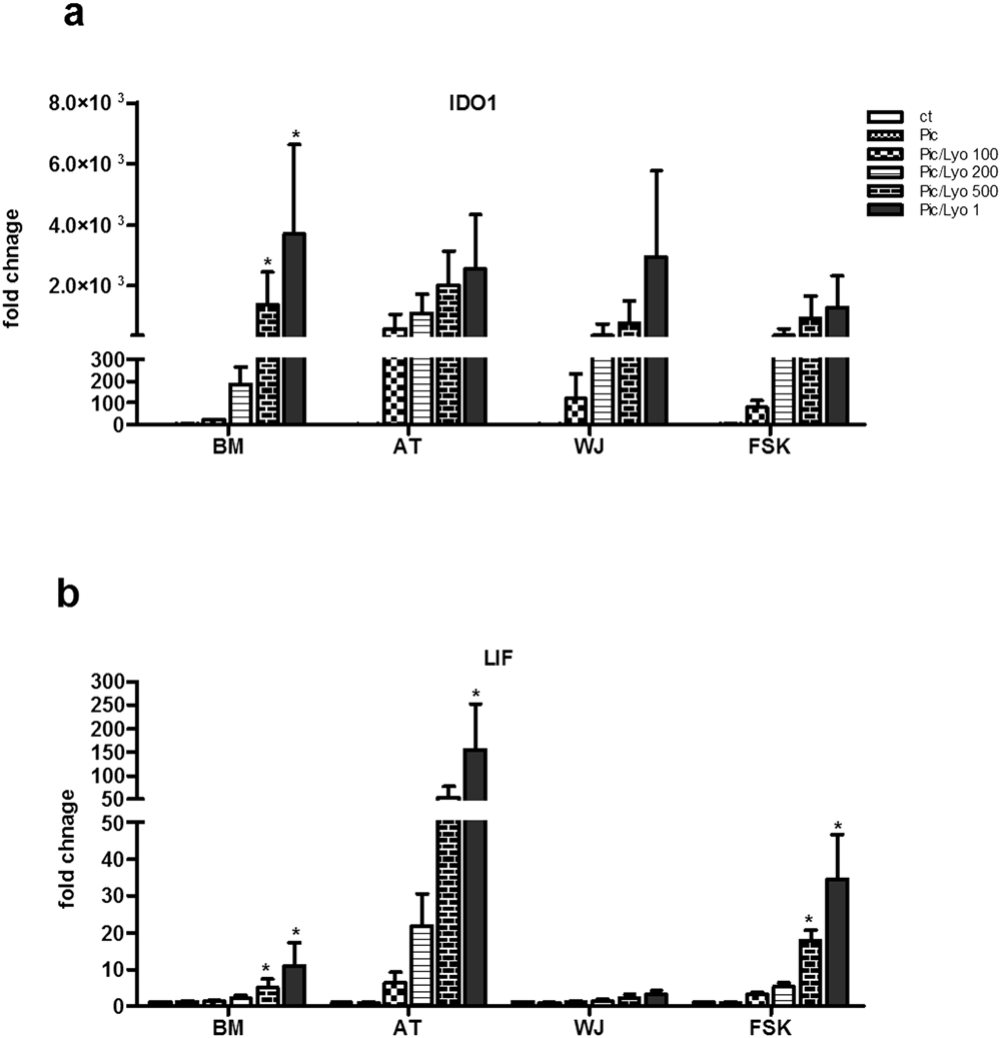
Activation of MDA-5 and RIG-I receptors in MSCs increases the expression of IDO and LIF. MSCs isolated from BM, AT, WJ and FSK were incubated in the medium in the presence of 25 µg/ml Poly(I:C) or were transfected with 100 ng/ml, 200 ng/ml, 500 ng/ml and 1 µg/ml of Poly(I:C)/LyoVec for 24 h. Then mRNA was extracted and the expression of (**a**) IDO and (**b**) LIF was evaluated by real time PCR. The results are expressed as relative mRNA fold change ± SEM (n = 3), *P < 0.05. Abbreviations: ctrl, control; Pic, Poly(I:C); Pic/Lyo, Poly(I:C)/LyoVec.

Immunoregulatory molecules such as LIF and IDO1 are mainly involved in the immunomodulatory effects of MSCs. Activation of RIG-I/MDA-5, induced different expression of LIF and IDO1 by MSCs according to their origin. IDO1 expression was induced in all MSC types by Poly(I:C)/LyoVec but not Poly(I:C) alone. However, differences among MSCs isolated from different sources were observed. AT-MSCs were the most sensitive to the presence of RLR ligand as at the lowest ligand concentration (100 ng/ml) IDO1 was 590-times more expressed whereas BM-MSCs were less sensitive with an only 20-fold change (Fig. 10a). Differently from IDO1, LIF expression was strongly induced by dsRNA only in AT-MSCs and slightly in FSK. WJ-MSCs did not express LIF in the presence of the RLR ligand (Fig. 10b).

### RIG-I/MDA-5 receptor activation in MSCs does not influence the immunophenotype but affects cell survival

To test if the MSC phenotype was influenced by RLR activation, we incubated studied cells for 24 h with a medium supplemented with Poly(I:C) or Poly(I:C)/LyoVec using the highest concentration. Flow cytometry data showed no changes in the expression of CD105, CD73, CD90, CD45, CD19, CD34, CD14 or HLA-DR in any of tested MSC types (Supplementary Table S2).

**Figure 11:**
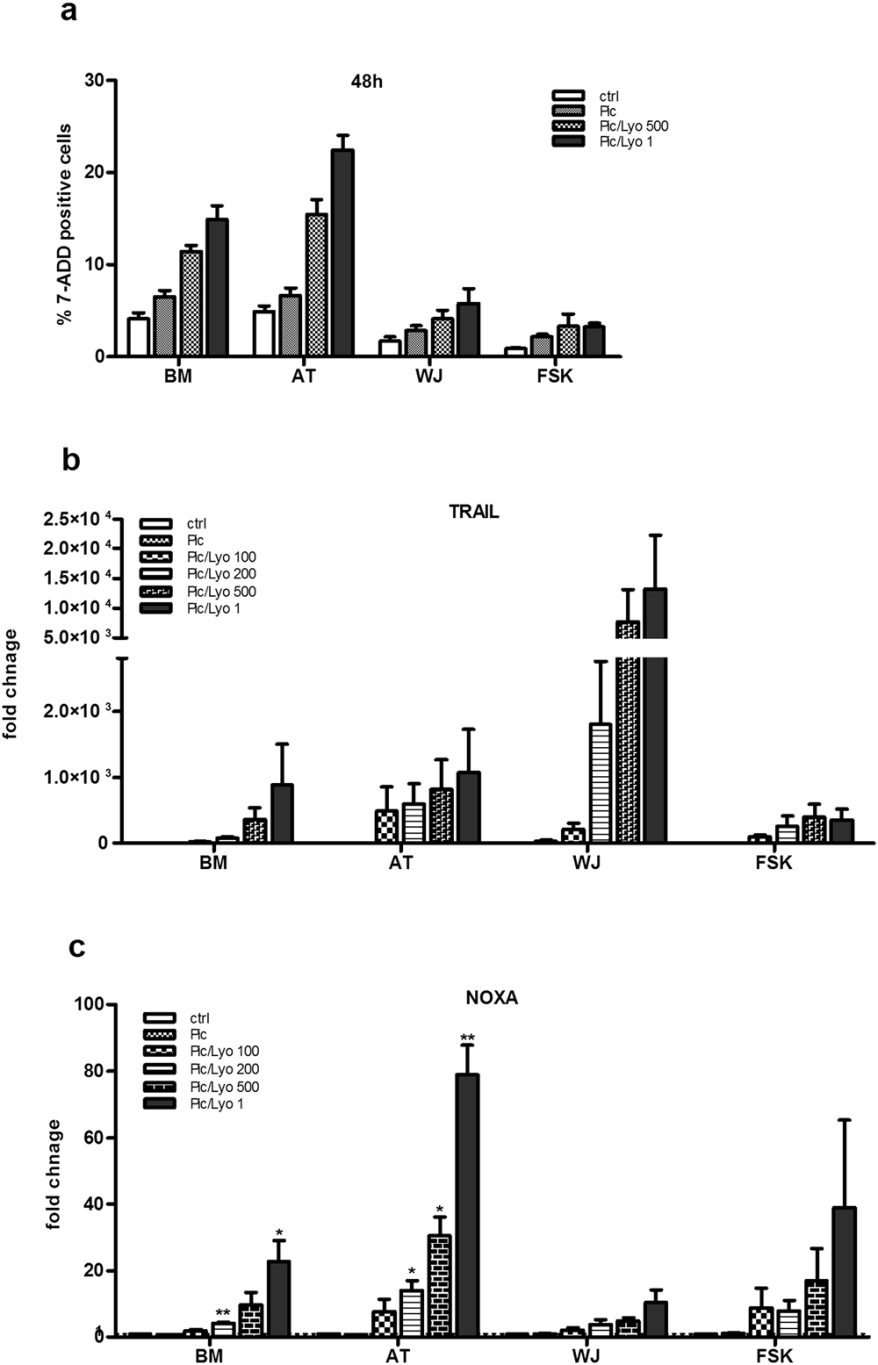
Activation of MDA-5 and RIG-I receptors in MSCs triggers the TRAIL and Noxa expression thus influencing cell survival. MSCs isolated from BM, AT, WJ and FSK were incubated in the presence of 25 ng/ml of Poly(I:C) or were transfected with 100 ng/ml, 200 ng/ml, 500 ng/ml or 1 µg/ml of Poly(I:C)/LyoVec. (**a**) After 48 h, the cells were stained with 7AAD and the late apoptotic cells were visualised using flow cytometry, n = 3. (**b,c**) After 24 h of incubation in the presence of different RLR ligands, mRNA was extracted from MSCs and the expression of (**b**) TRAIL and (**c**) Noxa was evaluated using real time PCR. The results are expressed as relative mRNA fold change ± SEM (n = 3), *p < 0.05, **p < 0.006. Abbreviations: ctrl, control; Pic, Poly(I:C); Pic/Lyo, Poly(I:C)/LyoVec.

In a parallel experiment, we tested the influence of different dsRNA concentrations on the apoptosis of studied MSC by measuring the Annexin V and 7AAD using flow cytometry. After 24 h of cell incubation in the presence of the RLR and TLR3 ligands we did not observe any important apoptotic process in any MSC type (data not shown). However, after 48 h of incubation, the MSC transfection with the two higher dsRNA concentrations increased the percentage of 7AAD labeled AT- and BM-MSCs cells without having any significant impact on the WJ- and FSK-MSCs (Fig. 11a).

### TRAIL and Noxa mRNA expression are induced in all MSC types upon RLR activation

Activation of RLR receptors is related to the expression of TRAIL in DCs and macrophages. As MSCs have active RIG-I/MDA5 receptors, we checked for the potential expression of TRAIL by studied MSC upon activation with dsRNA, as initially MSCs are known as TRAIL negative cells. Indeed, our experiment showed that MSCs do not express constitutively the TRAIL molecule. However, in the presence of the Poly(I:C)/LyoVec, but not Poly(I:C) alone, TRAIL mRNA was induced in all MSC types with the strongest fold change in WJ-MSC and the lowest one in FSK-MSC (Fig. 11b). Interestingly, we were not able to detect any TRAIL protein neither by flow cytometry nor by ELISA although TRAIL exists as well in soluble form. By flow cytometry, we tested two antibodies: the first one (from eBioscience) did not work as even with our positive control (PBMC) we did not see any labeled cells. Using a second antibody (from R&D Systems), we obtained a nice positive control but our cells were negative for TRAIL expression (data not shown).

Differently from TRAIL, which is a molecule involved in the extrinsic apoptotic pathway, Noxa is a molecule involved in the intrinsic apoptotic process. Our real time PCR data showed that the expression of Noxa in the presence of dsRNA is strongly increased only in AT-MSCs at the highest concentration, modestly in BM- and FSK-, and weakly in WJ-MSCs (Fig. 11c).

## Discussion

MSC modulate both the innate and adaptive immune responses variably according to their microenvironment^23, 24^. They sense exogenous and endogenous danger signals through different TLRs and NLRs which regulate some of their functions^25, 26^.

MSCs have dual effects on the antiviral immune responses^15^. Up to date, nothing is known about the expression profile and role of RLRs in human MSCs isolated from different tissues. In this study, we show that RLRs, RIG-I and MDA-5, are constitutively expressed by BM-, AT-, WJ- and FSK-MSCs. The expression level varied considerably among these MSC types with the highest expression observed in FSK-MSCs. Furthermore, RIG-I, LGP2 and MDA-5 expression was significantly up-regulated in the presence of an inflammatory environment or upon receptors activation, and this increase was ligand dose-dependent in all MSC types except in FSK-MSCs. This selevtive modulation of RLR expression confers to MSCs the ability to sense intracellular viral infection, to promptly adapt their immunobiological profile and finally respond by performing the appropriate immunomodulatory effects. The increased expression of LGP2, observed following RLR activation, indicate that it could strengthen the antiviral responses mediated by RIG-I- and MDA5. Indeed, LGP2 functions as a positive regulator of RIG-I and MDA5 responses by facilitating virus recognition and type I IFN production^17^.

Then we assessed the impact of RLR triggering on the downstream signaling pathway and secretome of MSC. Cytokine profile showed that depending on their origin, MSCs respond differently to RLR triggering and in a ligand dose-dependent manner. The activation of these receptors induced the strongest response in FSK- and AT-MSCs by increasing the expression of pro-inflammatory cytokines and interferons as compared to the two other MSC types.

Activation of RIG-I and MDA-5 induce downstream signaling through the mitochondrial adaptor MVAS (IPS-1)^2^. MVAS further mobilizes different types of kinases, activating IRF3, IRF7 and NF-kB transcription factors which in the nucleus coordinate the expression of the genes encoding IFN I and III and pro-inflammatory cytokines, respectively^1, 2, 27^. In the presence Poly(I:C), which activates TLR3, none of the studied MSCs secreted constitutively IFN-α/β. However, when Poly(I:C) was introduced into the cytoplasm of MSCs, activating thus MDA-5 and RIG-I, the production of IFN-β was strongly induced. It was recently shown that dsRNA/LyoVec acts on RLR rather than on TLR3 in human bronchial epithelial cells^27, 28^. Recently, Yu L *et al*. reported that the activation of TLR3 and RIG-I by poly(I:C) induced the expression of type I interferons and antivirus proteins in adipose-derived MSC^22^. Our work showed that among different MSC types those derived from the adipose tissue were the most sensitive to dsRNA presence in the cytoplasm as even at the lowest ligand concentration, the production of IFN-β was quite high. AT-MSCs were also described to produce IFN-β when cultured at high density^29^. Type I interferons (IFNs) are a family of cytokines involved in the immunity against viruses and other intracellular infections^30^. The main function of IFNs is the induction of dozens of proteins that interfere with virus replication in order to restrict viral spread. They are also positively linked to the activation and expansion of lymphocytes or NK cells that are important for control of intracellular infections^31^. Moreover, upon RLRs activation, studied MSCs produced IFN-λ1. The highest production of this type III IFNs was observed in AT-MSCs, as being 20-times higher than in WJ-MSCs, disclosing the lowest production. Human IFN-λ1 and IFN-β genes have similar transcriptional regulation that is controlled by either IRF3 or IRF7, whereas IFN-α genes are mostly IRF7 dependent^32^. In our experiments, it seems that the activation of IRF7 is linked with the increase in IFN-β expression in all MSC types. The treatment of MSCs with Amlexanox, an inhibitor of TBK1 and IKK-ε, confirmed the involvement of these enzymes and IRF7 in the MSC response to Poly(I:C) transfection. Yu L *et al*. observed the phosphorylation of IRF3 in adipose-derived MSCs after poly(I:C) stimulation^19^. In our experiments, the phosphorylation of IRF3 was undetectable. The phosphorylation of IRF7 without IRF3 activation has been also reported after poly(I:C) challenging of vascular endothelial cells^33^.

IFN-β and IFN-λ1, even thought signaling through common pathways and inducing similar biological activities, bind to different receptors with a cell type-restricted pattern of IFN-lR expression^34, 35^. While all cell types express receptor for type I IFN, IFN-λ1 primarily acts on epithelial cells and subsets of immune cells expressing IFN-lR1^36^. Comparing all four MSC types, we show that AT-MSCs are the strongest inducers of an antiviral response by secreting type I and type III IFN.

The activation of RLRs may also induce the expression of pro-inflammatory cytokines as shown in dendritic cells (DCs)^37^. In our experiment, we observed, a much lower up-regulation of pro-inflammatory cytokines as compared with IFN production. Nevertheless, when comparing the different MSC types, we observed that up-regulation of IL-6 and CCL5 mRNA was the strongest in FSK-MSCs. On the other hand, whereas the expression of IL-8 was increased in FSK- and AT-MSCs, and at very low level in BM-MSCs only at the highest dsRNA concentration, the expression of this chemokine was not influenced in WJ-MSCs by dsRNA presence. CCL5 and IL-8 are pro-inflammatory chemokines involved in the migration and chemotaxis of different immune cells^38^. Our data suggest that, when activated by RLR ligation, AT-MSCs induce the strongest direct antiviral response whereas FSK-MSCs respond by mounting an inflammatory reaction to fight against the viral presence.

The stimulation of RLRs could trigger both, the extrinsic and intrinsic pathways of cell death via BH3-only protein Noxa^18^. Recently, Yang *et al*. showed that in mouse MSCs, activation of RLRs induced both extrinsic and intrinsic apoptosis and suggested that RLR signaling is critical in controling MSC survival^21^. Thus, by using AnnexinV/7AAD double staining, we analysed the viability of our different MSC populations following transfection with Poly(I:C)/LyoVEC. Interestingly, only prolonged RLRs activation decreased the survival of AT-MSCs without having any impact on the viability of BM-, WJ- and FSK-MSCs. Indeed, indepedently of their origin, MSCs showed no significant cell death after 24 h of RLR activation. However, after 48 h of activation, the viability of AT-MSCs and, to lesser extent, of BM-MSCs was decreased. Afterwards, we investigated the mechanism underlying this cell death by checking the expression of TRAIL and Noxa recognized to be involved in the extrinsic and intrinsic apoptotic pathways respectively. MSCs are not known to express TRAIL but are considered as promising tool in cancer therapy when transfected with TRAIL-encoded plasmid^39, 40^. We observed that RLR activation in MSCs induced the expression of TRAIL mRNA and that expression was ligand dose and MSC-type dependent, whit the highest expression observed in WJ-MSCs. However, we did not detect TRAIL at the protein level even when we tested two different anti-TRAIL antibodies. Similar data were observed for Noxa mRNA expression. As the strongest expression of Noxa is found in AT-MSCs, it could explain why the viability of AT-MSCs is compromised in the presence of dsRNA.

As previously discussed^8, 9, 11^, the surrounding microenvironment determines the fate of MSCs and accordingly induce one of the two different MSC immunomodulatory phenotypes. IDO1 and LIF are among the regulatory mediators critically involved in MSC immunomodulation^41, 42^. We observed that none of studied MSCs expressed IDO1. However, in response to RLR activation, MSCs differently induced transcription of IDO1. This induction was ligand dose-dependent with the strongest and consistent IDO1 transcription observed in AT-MSCs. On the other side, the expression of LIF is also dependant of MSC origin. In a ligand dose-dependent manner, RLR activation strongly increased the expression of LIF in AT-MSCs and lo a lesser extent in FSK-MSCs. Following RLR activation, the increase of IDO1 and LIF expressions indicate an increase in immunodulatory function for some type of MSCs such as AT-MSCs.

Finally, as MSCs have a specific immunological profile which could be strongly affected by environmental conditions^43^, we studied the influence of RLR activation on their immunophenotype. In our experiments, wathever the source of MSCs, RIG-I and MDA-5 activation did not have any influence on the phenotype of these cells including their immunogenicity. This is of high interest when allogeneic MSCs are considered for cellular therapy.

In conclusion, our data show that MSCs isolated from BM, AT, WJ and FSK express functional RIG-I and MDA-5 receptors. Activation of these receptors, induce a tissue-specific and ligand dose-dependent modulation of MSC secretome as well as of their downstream signaling pathway. Among studied MSC typas, activation of RLRs induce the strongest response in AT-MSC. These MSCs secrete high amount of IFN-β and IFN-λ1 what could be linked with the increase in IRF7 transcription. Finally, in parallel with their proinflammatory phenotype, RLR activation by increasing the IDO1 and LIF expression enhances the immunomodulatory potential of AT-MSCs. Despite a slight decrease of AT-MSCs viability, all studied MSCs stayed in compliance with ISCT criteria and most importantly they preserved their low immunogenicity state. Altogether, among four studied MSC types, it appears that AT-MSCs are the most prominent one for mediating antiviral recognition and triggering consequent antiviral immune response. These data should be considered when designing MSC based immunotherapy in clinical setting where inflammation or infections are to be faced.

## Methods

### Isolation and cell culture

All samples were collected after approval by the local ethics committee of Bordet Institute and according to the principles expressed in the Declaration of Helsinki. The methods were carried out in accordance with the approved guidelines. Informed written consent was obtained for all donors.

MSCs were isolated from bone marrow, umbilical cord, adipose tissue, and foreskin as previously described (n = 3)^6, 44–46^. Briefly, mononuclear cells from BM were isolated by density gradient centrifugation (Biocoll, Biochrom, Berlin, Germany), washed in HBSS (Lonza Europe, Verviers, Belgium) and seeded at 2 × 10^4^ cells/cm^2^ in DMEM-LG (Lonza) supplemented with 15% of FBS (Sigma-Aldrich, Bornem, Belgium), 2 mM of L-glutamine, 50 U/ml of penicillin and 50 µg/ml of streptomycin (all from Lonza). After 48 hours, non-adherent cells were removed by washing. Umbilical cords from full-term deliveries were collected and umbilical cord segments were sectioned longitudinally to expose the Wharton Jelly’s on the plastic surface. MSCs were isolated based on their migratory and adhesive properties. AT was obtained from patients undergoing liposuction procedure. Lipoaspirates were washed with Dulbecco’s Phosphate-Buffered saline (DPBS, Lonza), and the extracellular matrix was digested with 0.1% collagenase D (Roche Applied Science, Vilvoorde, Belgium) at 37 °C for 30 min. After centifugation at 800 g for 10 min in complete culture medium, the stroma-vascular fraction pellet was cultured. FSK samples were obtained as discarded material from routine circumcision. FSK was sectioned longitudinally to spread the tissue and epidermis was manually removed from the skin. The dermis was then cut into small pieces and then treated by 0.2 mg/ml of liberase solution (Roche Diagnostics). After washing, the cell pellet was put in the culture^47^.

For all MSCs, when subconfluency (80–90%) was achieved, adherent cells were detached with TrypleSelect (Lonza) and expanded by replating at lower density (1000 cells/cm^2^). MSC were generally evaluated between 2 and 8 passages.

To confirm the mesenchymal nature of cells, CFU-F assay, phenotype and differentiation assays were performed as described^9, 48^.

### Reagents

The inflammatory environment was mimicked by using a cocktail of cytokines as previously described^9^: IL-1β 25 ng/ml (PeproTech, Rocky Hill, NJ), IFN-γ 10^3^ U/ml, TNF-α 50 ng/ml, and IFN-α 3 × 10^3^ U/ml (all from Prospec, Rehovot, Israel). The ligand for TLR3, Poly(I:C) and the ligand for MDA-5/RIG-I, Poly(I:C)/LyoVec were purchased from Sigma Aldrich (Diegem, Belgium) and InvivoGen (San Diego,CA,USA) respectively. Amlexanox, an inhibitor of the protein kinases TBK1 and IKK-ε, was purchased from InvivoGen and used at the concentration of 100 µM.

### RNA extraction and real-time PCR

Total mRNA was isolated from BM-, WJ-, FSK- and AT-MSCs using the TriPure Isolation Reagent (Roche Applied Science, Vilvoorde, Belgium). cDNAs was obtained by reverse transcription of 1 µg mRNA using qScript™ cDNA SuperMix (QUANTA BioSciences, Gaithersburg, MD, USA) for 5 min at 25 °C, 30 min at 42 °C and 5 min at 85 °C. qPCR was performed on an ABI Prism 7900HT Sequence Detection System (Applied Biosystem). We used 25 ng of cDNA in a qPCR with SYBR Green PCR Master Mix (Applied Biosystems, Rotterdam, The Netherlands) and 0.32 µM of gene-specific forward and reverse primers. The primers sequences used in the study are described in the Supplementary Table S1. We analyzed the data using the comparative threshold cycle method. The gene expression values were normalized to those of GAPDH and data were presented as mRNA levels relative (fold change) to the control (without receptor activation and defined as 1).

### Flow cytometry analysis

#### Phenotype

BM, AT, WJ and FSK-MSCs from three different donors were activated or not with 25 µg/ml of Poly(I:C) and 1 μg/ml of Poly(I:C)/LyoVec for 24 h and examined for the expression of different hematopoietic, endothelial and stromal cell surface markers using fluorochrome-conjugated monoclonal antibodies: CD34 (Becton, Dickinson, and Company, Franklin Lakes, NJ, USA); CD45 (Miltenyi Biotec, Leiden, the Netherlands), CD73, CD105 (Ancell Corporation, Bayport, MN, USA), CD90 (R&D Systems, Minneapolis, MN, UK), CD14, CD19 and HLA-DR (Beckman Coulter, Indianapolis, IN, USA).

#### Apoptosis and TRAIL expression

The MSCs isolated from four different sources (4 × 10^4^) were activated or not with 25 ng/ml of Poly(I:C) (TLR3) and 100 ng/ml, 200 ng/ml, 500 ng/ml and 1 μg/ml of Poly(I:C)/LyoVec (RIG-I/MDA5) for 24 h and 48 h. The cells were collected and re-suspended in Annexin V Binding buffer (Invitrogen, Gent, Belgium) and stained with Annexin V-FITC (early apoptosis) and nucleic acid dye 7-AAD (late apoptosis) (BD Biosciences, Erembodegem, Belgium) for 15 minutes in the dark.

Two antibodies for TRAIL protein were tested: monoclonal anti-human TRAIL(CD253)-PE (eBioscience, Vienna, Austria) and anti-human TRAIL/TNSF10-PerCP (R&D Systems).

#### RLR and TLR3 expression

TLR3 expression was assessed using anti-TLR3-PE (eBioscience) as previously reported^9^. For the intracellular detection of RIG-I and MDA-5 at the protein level in our four different MSC types, unlabelled rabbit anti-human polyclonal antibodies against RIG-I (IgG) and MDA-5 (IgG) were used (Abcam, Cambrige, UK). The cells were first fixed, permeabilized with Fix & Perm cell permeabilization kit (Imtec, Antwerpen, Belgium) and then incubated with appropriate primary antibodies for 30 min in the dark. After extensive wash with PBS (Lonza) the cells were stained with FITC-conjugated goat anti-rabbit IgG (H&L) polyclonal antibody (Abcam) for 1 h.

#### Activation of IRF3 and IRF7

The phosphorylation of IRF3 and IRF7 was evaluated by flow cytometry. MSCs were fixed and permeabilized using the Cell Signaling Buffer Set A (Miltenyi Biotec). Cells were then stained with anti-phospho-IRF3 (Ser396)-PE (Cell Signaling Technology) and anti-IRF7 pS477/pS473 PE antibodies (Miltenyi Biotec) during 30 min.

Flow cytometry (FC) data acquisition was realized on a MacsQuant analyzer (MACS Miltenyi Biotec) and the analysis performed with FCS 4 Express software (DeNovo Software).

### Cytokine production

After activation of TLR3 and RIG-I/MDA5 in different types of MSCs with Poly(I:C) and Poly(I:C)/LyoVec for 24 h, culture supernatants were collected and the levels of IFN-β and IFN-λ1 (IL-29) were measured by ELISA (R&D Systems, Abingdon, United Kingdom; eBioscience, Vienna, Austria).

### Transfection of small interfering RNA (siRNA)

Small interfering RNA (siRNA) targeting RIG-I (sc-61480), TLR-3 (sc-36685) and control siRNA targeting a scrambled sequence were purchased from Santa Cruz Biotechnology. Transfection of siRNA was performed using Lipofectamine RNAiMAX (Life Technologies) according to the manufacturer’s instructions. MSCs seeded in six-well plates were transfected with 100 nM siRNA in serum-free medium and after 24 h, the cells were stimulated with Poly(I:C)/LyoVec at 1 µg/ml.

### Statistical analysis

Statistical differences were evaluated by a two-tailed or, when applicable, one-tailed paired t-test or Mann-Withney U test using GraphPad Prism version 5.00 for Windows (GraphPad Software, San Diego California USA, www.graphpad.com). P values less than 0.05 were considered as statistically significant.

## Electronic supplementary material

Dataset 1
