# Supplementary material for: Comparison and immunobiological characterization of retinoic acid inducible gene-I-like receptor expression in mesenchymal stromal cells
Source: Sci Rep. 2017 Jun 6;7:2896. doi: 10.1038/s41598-017-02850-6 (PMC5460162; doi:10.1038/s41598-017-02850-6)
Supplement: Supplementary file 1 — Dataset 1 [file 41598_2017_2850_MOESM1_ESM.doc]

**Supplementary informations**

**COMPARISON AND** **IMMUNOBIOLOGICAL CHARACTERIZATION OF RETINOIC ACID INDUCIBLE GENE-I-LIKE RECEPTOR EXPRESSION IN** **MESENCHYMAL STROMAL CELLS**

Gordana Raicevic^1*^, Mehdi Najar^1*^, Hélène Busser^1^, Emerence Crompot^1^, Dominique Bron^1,2^, Michel Toungouz^1,3^, Laurence Lagneaux^1^

(1) Laboratory of Clinical Cell Therapy, Institut Jules Bordet, Université Libre de Bruxelles; (2) Department of Hematology, Jules Bordet Institute;  (3) Department of Immunology-Hematology-Transfusion, Hôpital Erasme, Université Libre de Bruxelles, Brussels, Belgium.

*Supplementary Table S1. Sequences of real-time PCR primers used in the study*.

| Genes | Forward 5’→ 3’ | Reverse 5’→ 3’ |
| --- | --- | --- |
| *GAPDH* | GCAGGATTTGTAAAGCCCTGTT | CACTGATAATGAGGGCATCATTATATTT |
| *RIG-I (DDX58)* | GCAGGATTTGTAAAGCCCTGTT | CACTGATAATGAGGGCATCATTATATTT |
| *MDA-5* | AGCATCTGAGCCTGGAAAAGTTA | CTTGCGGAAGAGCTGTTCAAC |
| *LGP-2* | TCATCTGTACGGCAGAGTTGT | TGTTGTAGACGGTGTCCTTGT |
| *TLR3* | TTAAAGAGTTTTCTCCAGGGTGTTTT | AATGCTTGTGTTTGCTAATTCCAA |
| *IFN-β,* | AGCTGAAGCAGTTCCAGAAG | AGTCTCATTCCAGCCAGTGC |
| *IFN-λ1(IL-29)* | GTGGTGCTGGTGACTTTGG | CTCCTGTGGTGACAGAGATTTG |
| *CCL5* | TCTGCGCTCCTGCATCTG | GGGCAATGTAGGCAAAGCA |
| *IL-6* | AAATTCGGTACATCCTCGACGG | GGAAGGTTCAGGTTGTTTTCTGC |
| *IL-8* | CTGTTAAATCTGGCAACCCTAGTCT | CAAGGCACAGTGGAACAAGGA |
| *TNF-a* | ATCTTCTCGAACCCCGAGTGA | AGCTGCCCCTCAGCTTGA |
| *IRF3* | ACACATACTGGGCAGTGAGC | GCACAACCTTGACCATCACG |
| *IRF5* | CGGACTGATGTGGAGATGTG | CTCTCCTTCTTGGCCCAAAT |
| *IRF7* | TACCATCTACCTGGGCTTCG | AGGGTTCCAGCTTCACAG |
| *Noxa* | GCTGGAAGTCGAGTGTGCTA | CCTGAGCAGAAGAGTTTGGA |
| *TRAIL* | ACCAACGAGCTGAAGCAGAT | CAAGTGCAAGTTGCTCAGGA |
| *IDO1* | TTCAGTGCTTTGACGTCCTG | TGGAGGAACTGAGCAGCAT |
| *LIF* | TGAAAACTGCCGGCATCTGA | CTGTGTACTGCCGCCAAGA |

Supplementary Table S2. *Phenotype of different MSC types when activated or not*

*with TLR3 and RLR ligands.*

| **Marker** | **Condition** | **BM-MSC**  **(%)** | **AT-MSC**  **(%)** | **WJ-MSC**  **(%)** | **FSK-MSC**  **(%)** |
| --- | --- | --- | --- | --- | --- |
| **CD105** | ctrl  PIC  PIC/Lyo | 94,1 ± 1,6  90,9 ± 1,2  93,3 ± 0,8 | 94,2 ± 2,1  94,4 ± 1,5  95,2 ± 3,6 | 79,7 ± 3,3  83,5 ± 3,8  83,4 ± 4,1 | 92,9 ± 0,5  91,0 ± 1,5  91,9 ± 0,9 |
| **CD73** | ctrl  PIC  PIC/Lyo | 99,1 ± 0,4  97,7 ± 0,3  97,5 ± 0,5 | 97,4 ± 2,0  99,5 ± 0,2  96,7 ± 2,4 | 97,2 ± 0,3  97,9 ± 0,6  98,9 ± 0,4 | 99,5 ± 0,3  99,7 ± 0,1  98,9 ± 0,3 |
| **CD90** | ctrl  PIC  PIC/Lyo | 93,1 ± 1,7  91,7 ± 2,5  91,3 ± 1,7 | 97,0 ± 0,7  96,9 ± 0,6  95,6 ± 1,2 | 97,3 ± 0,8  94,9 ± 1,5  95,8 ± 0,6 | 99,0 ± 0,4  98,9 ± 0,5  97,3 ± 0,6 |
| **HLA-DR** | ctrl  PIC  PIC/Lyo | 1,1 ± 0,4  2,8 ± 0,2  2,4 ± 0,6 | 2,4 ± 0,5  2,5 ± 0,8  2,1 ± 0,5 | 1,9 ± 0,7  2,1 ± 0,6  2,0 ± 0,3 | 2,4 ± 0,6  1,8 ± 0,4  1,7 ± 0,4 |
| **CD45** | ctrl  PIC  PIC/Lyo | 2,2 ± 0,3  1,7 ± 0,3  2,2 ± 0,5 | 2,9 ± 1,0  3,7 ± 0,8  2,9 ± 0,5 | 2,2 ± 0,5  2,2 ± 0,4  2,6 ± 0,4 | 3,4 ± 0,8  3,1 ± 0,4  4,0 ± 0,7 |
| **CD19** | ctrl  PIC  PIC/Lyo | 2,3 ± 0,7  2,5 ± 0,5  2,3 ± 0,3 | 2,2 ± 0,6  2,1 ± 0,3  1,7 ± 0,3 | 2,2 ± 0,3  1,9 ± 0,4  1,4 ± 0,3 | 2,7 ± 0,8  2,8 ± 0,5  3,0 ± 0,9 |
| **CD14** | ctrl  PIC  PIC/Lyo | 0,6 ± 0,2  1,0 ± 0,3  1,2 ± 0,2 | 1,2 ± 0,4  1,5 ± 0,6  1,4 ± 0,6 | 1,4 ± 0,5  1,5 ± 0,5  1,4 ± 0,4 | 2,8 ± 1,1  1,9 ± 1,4  2,8 ± 0,9 |
| **CD34** | ctrl  PIC  PIC/Lyo | 2,4 ± 0,6  2,6 ± 1,2  3,9 ± 0,7 | 3,4 ± 0,7  3,0 ± 0,6  3,7 ± 0,4 | 2,3 ± 1,0  2,9 ± 0,7  3,2 ± 0,3 | 2,6 ± 0,7  2,5 ± 0,8  3,6 ± 1,2 |

Values represent percentage (%) of cells expressing the specific marker ± standard error of the mean. For each source of MSC three different donors were used (n=3). Abbreviations: ctrl, control; PIC, Poly(I:C); PIC/Lyo, Poly(I:C)/LyoVec.
